# Long‐term effects of influenza and Bacille Calmette–Guérin vaccination on systemic inflammation

**DOI:** 10.1002/cti2.70047

**Published:** 2025-09-11

**Authors:** Priya A Debisarun, Rutger J Röring, Özlem Bulut, Thijs ten Doesschate, Thomas W van der Vaart, Vinod Kumar, Helga Lemmers, Heidi Dijkstra, Axel B Janssen, Karin Veerman, Rob ter Heine, Reinout van Crevel, Jaap ten Oever, Leo AB Joosten, Marc J Bonten, Cornelis H van Werkhoven, Janneke HHM van de Wijgert, Mihai G Netea

**Affiliations:** ^1^ Department of Medicine and Radboud Center for Infectious Diseases Radboud University Medical Center Nijmegen The Netherlands; ^2^ Department of Medical Microbiology, Infectious Diseases & Infection Prevention Maastricht University Medical Center Maastricht The Netherlands; ^3^ Inflammatory Origins Group IIGH Theme, Murdoch Children's Research Institute, Royal Children's Hospital Parkville VIC Australia; ^4^ Julius Center for Health Sciences and Primary Care University Medical Center Utrecht, University of Utrecht Utrecht The Netherlands; ^5^ Department of Infectious Diseases Amsterdam University Medical Center, University of Amsterdam Amsterdam The Netherlands; ^6^ Department of Genetics University Medical Center Groningen Groningen The Netherlands; ^7^ Department of Medical Microbiology University Medical Center Utrecht, University of Utrecht Utrecht The Netherlands; ^8^ Department of Internal Medicine Sint Maartenskliniek Nijmegen The Netherlands; ^9^ Department of Pharmacy, Research Institute for Medical Innovation Radboud University Medical Center Nijmegen The Netherlands; ^10^ Department of Medical Genetics Iuliu Hatieganu University of Medicine and Pharmacy Cluj‐Napoca Romania; ^11^ Department for Immunology and Metabolism, Life and Medical Sciences Institute (LIMES) University of Bonn Bonn Germany

**Keywords:** Bacille Calmette–Guérin, inflammatory biomarkers, influenza vaccine, low‐grade inflammation, SARS‐CoV‐2, trained immunity

## Abstract

**Objective:**

Chronic systemic inflammation can lead to metabolic, cardiovascular and neurodegenerative complications, but the factors influencing it are incompletely understood. In this study, we evaluated several factors, including Bacille Calmette–Guérin (BCG) and influenza vaccination, SARS‐CoV‐2 infection and sex, that may impact systemic inflammation as assessed by targeted inflammatory plasma proteome analysis in healthy individuals.

**Methods:**

Participants were randomised to BCG or placebo vaccination at the start of the Dutch SARS‐CoV‐2 epidemic in March/April 2020. They reported their influenza vaccination status for the most recent influenza season. Twelve weeks after BCG or placebo vaccination, we assessed relative concentrations of 69 proteins in plasma of 357 individuals.

**Results:**

Both BCG and quadrivalent influenza vaccination were associated with overall trends towards reduced systemic inflammation in both sexes, but with a more pronounced effect in men. However, the impact on specific immunological proteins varied between BCG and influenza vaccinations. SARS‐CoV‐2 infection in the 12 weeks between randomisation and plasma sampling was also associated with overall trends towards reduced systemic inflammation, reaching significance for CXCL10 and TNF concentrations. Notably, individuals who had received BCG vaccination prior to SARS‐CoV‐2 infection did not exhibit this protein profile. Furthermore, elevated CXCL11 and OPG concentrations at 12 weeks were associated with subsequent respiratory symptoms during the additional 9 months of follow‐up.

**Conclusions:**

Our study revealed distinctive alterations in the plasma inflammation proteome associated with BCG vaccination, influenza vaccination, SARS‐CoV‐2 infection and sex. These findings are exploratory and hypothesis‐generating and warrant further investigation in well‐controlled longitudinal cohort studies.

## Graphical abstract

Plasma proteins were assessed in 357 participants, revealing that both BCG and influenza vaccinations were associated with decreased systemic inflammation, particularly in men. However, proteomic changes were different for influenza and BCG. Vaccination with both influenza and BCG had no additive effects on systemic inflammation. These exploratory findings warrant validation in a larger follow‐up study.

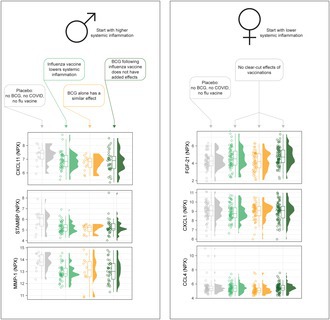

## Introduction

Low‐grade chronic inflammation is involved in the pathophysiology of many diseases, including metabolic syndrome, cardiovascular and neurodegenerative diseases.^1^, ^2^ Many factors have been described to influence systemic inflammation, including advanced age, obesity, diet, microbiome, as well as various inflammatory and infectious diseases.^3^, ^4^, ^5^ One factor that received little attention for its effect on systemic inflammation is vaccination. Vaccines have traditionally been given to children, and they protect against target diseases through specific antigen‐dependent antibodies and T cells.^6^ However, in the last decades, we have witnessed the introduction of vaccination in other groups at high risk for infections, such as the elderly, in whom chronic inflammation plays an important role in the establishment of comorbidities.^7^, ^8^ Moreover, new insights into the immunological mechanisms induced by vaccines have demonstrated effects on innate immunity and inflammation, beyond the specific effect on the target infection.^9^ Indeed, it has been recently demonstrated that certain vaccines, such as Bacille Calmette–Guérin (BCG) increase heterologous protection beyond the target mycobacterial infections, through a process of functional and epigenetic reprogramming of innate immune cells termed *trained immunity* or innate immune memory.^10^, ^11^, ^12^, ^13^

In addition to its capacity to increase antimicrobial properties of innate immune cells, our group has recently shown that BCG can also decrease systemic inflammation in a cohort of mostly young individuals.^14^ If further validated in additional studies, this finding could have important consequences for the prophylaxis of inflammatory disorders. Moreover, this also raises the question of whether other vaccines, especially those administered to older individuals, can modify the circulating inflammatory profile. Given the widespread use of influenza vaccines in the elderly, studying its potential impact on systemic inflammation is essential.^15^

In the present study, we aimed to explore factors that impact plasma inflammation markers in healthy individuals, with a focus on BCG and influenza vaccinations, as well as SARS‐CoV‐2 infection. For this, we took advantage of vaccination trials performed during the COVID‐19 pandemic. During the COVID‐19 pandemic, prior to the availability of SARS‐CoV‐2‐specific vaccines, several clinical trials were performed to assess the efficacy of BCG vaccination against SARS‐CoV‐2 infection through its heterologous effects.^16^, ^17^, ^18^, ^19^ In 2020–2021, we performed the BCG‐CORONA trial, in which the incidence and severity of SARS‐CoV‐2 infection in BCG vaccinated and placebo vaccinated Dutch healthcare workers was investigated.^20^ While no effect of BCG on the incidence of SARS‐CoV‐2 infection was observed, the impact on the incidence of COVID‐19 hospitalisation could not be assessed because of the limited number of cases in this cohort.^21^ The trial provided the opportunity to investigate the impact of BCG vaccination, as well as of influenza vaccination that is also recommended for healthcare professionals, on plasma inflammation markers. In addition, we analysed the association of circulating inflammatory biomarkers with SARS‐CoV‐2 and other respiratory infections.

## Results

### Baseline characteristics

We analysed plasma inflammation markers from 357 individuals, of whom 251 were women and 106 were men. Baseline characteristics of the study population can be found in Table 1.

**Table 1:** Population characteristics for the included participants, stratified by vaccination status

	Control^a^	BCG only	Flu vaccine only	Flu vaccine + BCG	*P* *
Participants (*n*)	66	69	110	112	—
Women (*n*, %)	55 (83.33%)	54 (78.26%)	65 (59.09%)	77 (68.75%)	2.56 × 10^−3^
Age (mean ± SD)	42.00 ± 13.77	41.07 ± 13.45	41.80 ± 12.42	41.74 ± 12.67	9.58 × 10^−1^
Positive COVID‐19 serology at 12 weeks (*n*, %)	1 (1.52%)	6 (8.70%)	3 (2.73%)	5 (4.46%)	1.54 × 10^−1^
Respiratory episode between plasma sampling (3 months after randomisation) and end of follow‐up (12 months after randomisation) (*n*, %)	6 (9.09%)	4 (5.80%)	11 (10.00%)	13 (11.61%)	5.14 × 10^−1^

^a^ Control group: people who had received neither BCG nor the influenza vaccine.

* The Fisher's exact test for categorical variables and the Kruskal–Wallis test for age.

### Factors influencing the inflammatory proteome in Dutch healthcare workers

**Figure 1:**
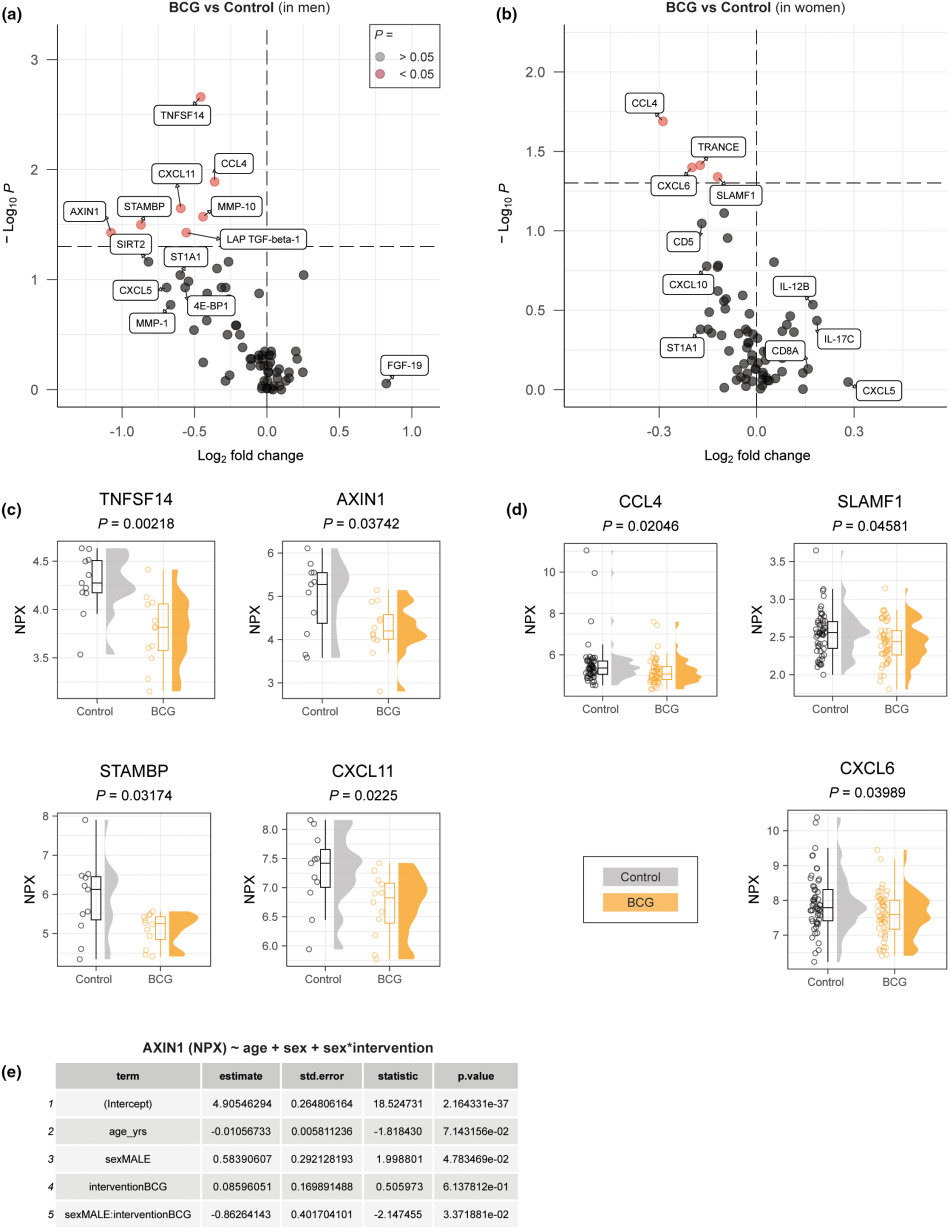
Effect of Bacille Calmette–Guérin (BCG) on systemic inflammation in men and women after 3 months. **(a)** A volcano plot of plasma inflammatory proteome (BCG vs control) in men. **(b)** A volcano plot of plasma inflammatory proteome (BCG vs control) in women. **(c)** Normalised protein expression (NPX) values of differentially circulating proteins in men. **(d)** NPX values of differentially circulating proteins in women. **(e)** Linear regression analysis of AXIN1. The NPX value was used as a continuous outcome variable and age, sex and vaccine exposures as independent predictor variables.

We assessed the effect of BCG and influenza vaccination, as well as SARS‐CoV‐2 and overall respiratory symptoms, on the plasma inflammation proteome. We first validated the known observation that men generally have higher systemic inflammation than women. Indeed, in control individuals nearly all analysed proteins had greater median NPX values in plasma samples from men than from women (Supplementary figure 1a). Older people also tended to have higher concentrations of circulating inflammatory proteins (Supplementary figure 1b). Notably, age was not different across vaccination status groups. Led by these observations, and because BCG is known to have differential effects in men and women,^14^ we performed sex‐stratified analyses. In men who received BCG vaccination only (*n* = 12), median NPX values of seven proteins from the inflammation panel were significantly lower, 37 non‐significantly lower and 25 non‐significantly higher, compared to men in the control group (*n* = 11). In women who received BCG vaccination only (*n* = 51), median NPX values of four proteins were significantly lower, 38 were non‐significantly lower, and 27 were non‐significantly higher, compared to women in the control group (*n* = 54) (Figure 1a and b). The BCG‐associated median NPX value difference for all proteins combined was larger in men (log_2_FD *P* = 1.86 × 10^−4^) than in women (*P* = 0.052), indicating a greater difference in overall levels of inflammatory proteins in men. TNFSF14 (a costimulatory factor for lymphocytes), STAMBP (a negative regulator of AKT–mTOR pathway), AXIN1 (a negative regulator of WNT signalling pathway) and CXCL11 (an interferon inducible chemoattractant for activated T cells) were the most strongly downregulated (the lowest log_2_FD among the proteins with *P* < 0.05) in men with BCG‐only compared to men in the control group (Figure 1c). CCL4 (a chemokine produced by activated immune cells) was significantly downregulated in the BCG‐only compared to control groups in both men and women, although with greater magnitude in men. Other proteins with mild, though statistically significantly lower median NPX values in women who received BCG‐only compared to controls were CXCL6 (chemoattractant for neutrophils) and SLAMF1 (important for T‐cell function), depicted in Figure 1d. We performed a linear regression analysis for AXIN1, the protein with the largest log_2_FD in men, but not in women (Figure 1e). The model revealed that, besides significant associations between NPX value and age and sex, a significant interaction effect was present between sex and BCG vaccination. These findings raise the possibility of a sex‐specific association between BCG vaccination and lower relative concentrations of plasma inflammation proteins.

### Effects of BCG vaccination on circulating inflammatory markers in SARS‐CoV‐2‐positive individuals

**Figure 2:**
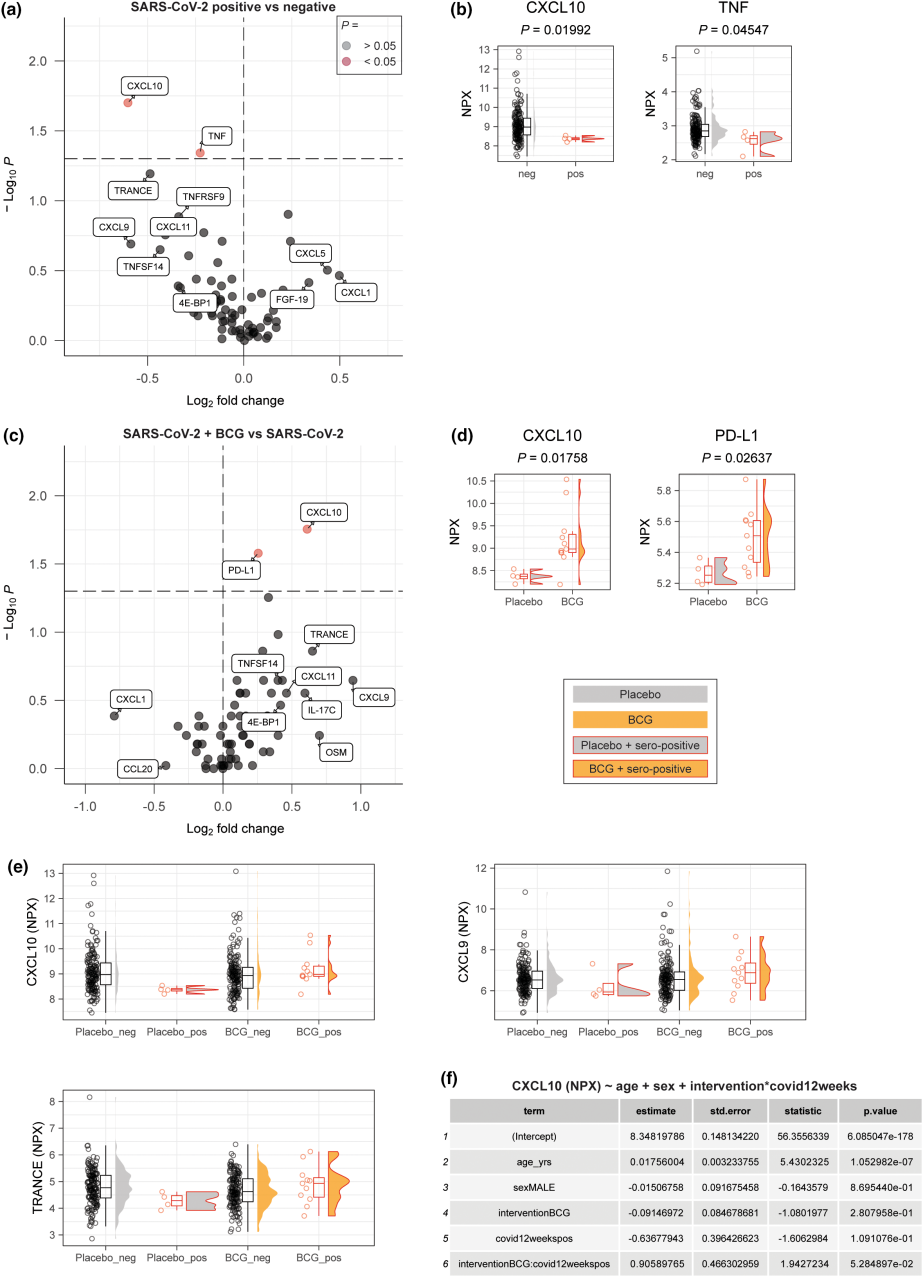
Effects of COVID‐19 on the circulating inflammatory proteome and interplay with prior Bacille Calmette–Guérin (BCG) vaccination. **(a)** A volcano plot describing the effects of SARS‐CoV‐2 infection on the inflammatory proteome in people who were randomised to placebo vaccination. **(b)** NPX values of top differentially circulating proteins in individuals with positive serology for SARS‐CoV‐2 but no prior BCG vaccination. **(c)** A volcano plot describing the effects of BCG vaccination in those individuals who became seropositive for SARS‐CoV‐2 in the 12 weeks following inclusion. **(d)** Top affected proteins by BCG. **(e)** NPX values of CXCL10, CXCL9 and TRANCE to illustrate the interaction of BCG and of SARS‐CoV‐2 on systemic inflammation. **(f)** Linear regression analysis of CXCL10. The NPX value was used as a continuous outcome variable and age, sex and vaccine exposures as independent predictor variables.

We compared the plasma inflammation proteome of individuals who had evidence of a SARS‐CoV‐2 infection in the 12‐week period between randomisation and sampling (*n* = 11 in the BCG group and *n* = 4 in the placebo group) to those who did not (*n* = 170 in the BCG group and *n* = 172 in the placebo group) (Supplementary figure 1a). Stratification of these analyses by sex and influenza vaccination status was not possible because of the low numbers of SARS‐CoV‐2 infections. SARS‐CoV‐2 infection was associated with a trend towards lower systemic inflammation markers in the placebo group (Figure 2a). In the SARS‐CoV‐2‐positive group (*n* = 4), the median NPX values of two proteins were significantly lower, 42 were non‐significantly lower, and 25 were non‐significantly higher, compared to the SARS‐CoV‐2‐negative group (*n* = 172). Specifically, CXCL10 (IP‐10, closely related to CXCL9 and CXCL11) and TNF were significantly downregulated in individuals with positive SARS‐CoV‐2 serology (Figure 2b). The combined median log_2_FD across all proteins was significantly below 0 (*P* = 0.012), indicating a general downward shift in protein expression levels in the SARS‐CoV‐2‐positive group. This suggests that, rather than isolated changes in individual proteins, there was a coordinated suppression across the broader proteomic profile. This finding was, however, based on small sample sizes, and thus firm conclusions remain elusive.

In the select subgroup of participants who developed SARS‐CoV‐2 infection within 12 weeks of randomisation, the protein signature differed markedly depending on whether they had received BCG or placebo. In the BCG group (*n* = 11), the downregulated protein signature observed in SARS‐CoV‐2‐infected placebo recipients (*n* = 4) was not seen (Figure 2c). Instead, the median NPX values of 21 proteins were non‐significantly lower, two significantly higher, and 46 were non‐significantly higher. The median log_2_FD for all panel proteins combined was significantly higher than 0 (*P* = 2.36 × 10^−4^). This suggests a broad upward shift in protein expression levels. The median NPX value of CXCL10—a chemokine associated with viral infections and interferon responses—was significantly lower in the SARS‐CoV‐2‐positive group, but this reduction was absent when BCG had been administered prior to infection; in addition, the median NPX value was higher for soluble PD‐L1 (a checkpoint‐protein related to T‐cell activation) (Figure 2d and e). We performed linear regression analysis for CXCL10 (the protein with the largest fold difference and *P* < 0.05 in Figure 2a), which suggested (*P* < 0.1; to be interpreted with caution) a significant interaction between BCG vaccination and SARS‐CoV‐2 infection (Figure 2f). These observations are intriguing, as they hint at a BCG‐mediated modulation of the host immune response to SARS‐CoV‐2, potentially consistent with trained immunity. However, because of small sample sizes and the exploratory nature of these analyses, these results require confirmation in larger, adequately powered studies before firm conclusions can be drawn.

### Effect of influenza vaccination on circulating inflammatory markers of healthcare workers

**Figure 3:**
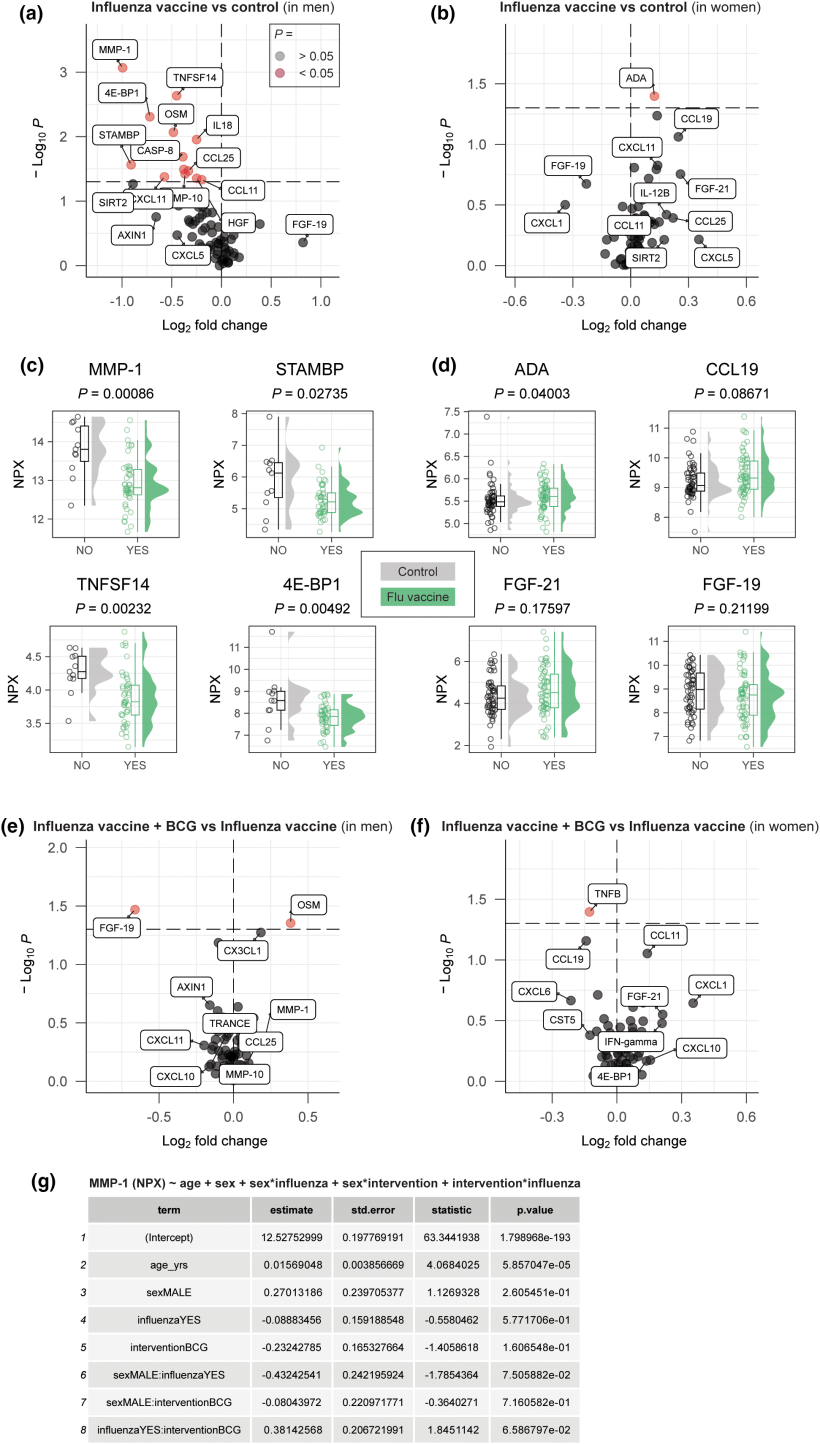
Effects of the influenza vaccine on systemic inflammation in men and women after 6–8 months. **(a)** A volcano plot showing the long‐term effects of the influenza vaccine on systemic inflammation in men and **(b)** in women. **(c)** Top hits of the previous volcano plots in men and **(d)** in women. **(e)** The additive effect of the Bacille Calmette–Guérin (BCG) vaccine on systemic inflammation in men and **(f)** in women who had previously received the influenza vaccine. **(g)** Linear regression analysis of MMP‐1. The NPX value was used as a continuous outcome variable and age, sex and vaccine exposures as independent predictor variables.

We also investigated the long‐term effects of influenza vaccination (the plasma sample was taken more than 6 months after the institutional influenza vaccination campaign) on the inflammation proteome. Men who received influenza vaccination only (*n* = 45) had a trend towards lower inflammation compared to men in the control group (*n* = 11; Figure 3a). Median NPX values were significantly lower for 13 proteins, non‐significantly lower for 36 proteins and non‐significantly higher for 20 proteins. The median log_2_FD for all proteins combined was significantly lower than 0 (*P* = 3.11 × 10^−6^). Women who received influenza vaccination only (*n* = 62), compared to women in the control group (*n* = 54), showed a reversed trend. Median NPX values were non‐significantly lower for 18 proteins, significantly higher for one protein (ADA) and non‐significantly higher for 50 proteins. The log_2_FD for all proteins combined was significantly higher than 0 (*P* = 7.75 × 10^−5^; Figure 3b). Among the 13 proteins significantly downregulated in men, were MMP‐1 (an important metallopeptidase involved in tissue remodelling), 4E‐BP1 (central in signalling pathways related to cell survival), as well as STAMBP and TNFSF14 (Figure 3c). The latter two also had significantly lower relative concentrations in men who received BCG vaccination (Figure 2a). In women who received influenza vaccination (*n* = 62), only the relative concentration of ADA (its deficiency is associated with lack of cellular and humoral immunity) was significantly higher although the effect size was very minor. FGF21 (a regulator of systemic glucose levels) and CCL19 (also known as MIP‐3‐β, shows chemotactic activity for T‐ and B cells) showed a non‐significant trend towards increase. Conversely, FGF‐19 concentrations were lower in women who had previously received an influenza vaccine (Figure 3d).

Men and women who received influenza and subsequent BCG vaccination had similar protein profiles as those who received influenza vaccination only (Figure 3e and f). We performed linear regression analysis for MMP‐1 (the protein with the largest difference in relative concentration in Figure 3a), which suggested interactions (*P* < 0.1; to be interpreted with caution) between sex and influenza vaccination, and between influenza vaccination and BCG vaccination (Figure 3g). These data suggest a similar association between influenza vaccination and plasma inflammation proteins as described above for BCG, while there was no such association for BCG in people who had already received the influenza vaccine. We speculate that this suggests BCG has no add‐on effect once influenza vaccination has occurred.

### The association between the inflammatory proteome and respiratory symptoms

**Figure 4:**
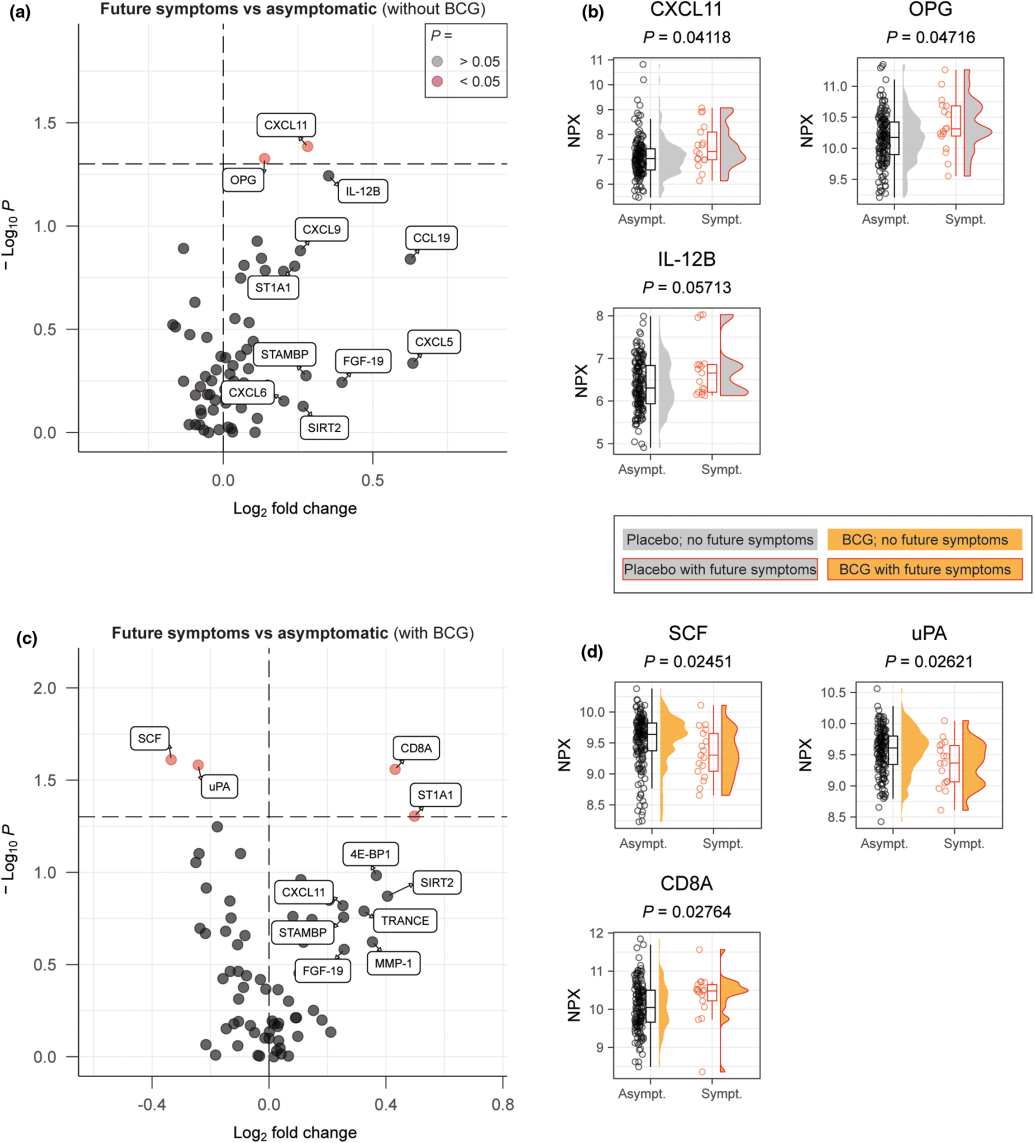
Predictive value of the circulating inflammatory proteome for developing respiratory symptoms in the following 9 months. **(a)** A volcano plot detailing the differences between those who developed respiratory symptoms during follow‐up vs those who did not in individuals randomised to placebo vaccination. **(b)** Top differentially circulating proteins from A. **(c)** A volcano plot detailing the differences between those who developed respiratory symptoms during follow‐up vs those who did not in individuals who were randomised to Bacille Calmette–Guérin (BCG) vaccination. **(d)** Top differentially circulating proteins from the previous volcano plot.

To assess whether the relative concentrations of specific plasma inflammation proteins were associated with subsequent respiratory symptoms, we evaluated the symptoms reported by participants in the 9 months following plasma sampling. The plasma inflammation proteome of individuals who reported respiratory symptoms was compared to the proteome of those who never reported any symptoms during follow‐up, stratified for BCG or placebo. In the placebo group, participants who reported respiratory symptoms (*n* = 17) had significantly higher median NPX concentrations of two proteins (CXCL11 and OPG), non‐significantly higher for 23 proteins and non‐significantly lower for 44 proteins than participants who remained symptom‐free (n = 159; Figure 4a and b); the overall median log_2_FD for all proteins together was higher than 0 (*P* = 8.83 × 10^−3^). In the BCG group, participants who reported respiratory symptoms (*n* = 17) had significantly higher median NPX values for two proteins (CD8A and ST1A1), non‐significantly higher for 34 proteins, significantly lower for two proteins (SCF and uPA) and non‐significantly lower for 31 proteins than participants who remained symptom‐free (*n* = 164); the overall median log_2_FD for all proteins combined was not significantly different from 0 (*P* = 0.96) (Figure 4c and d).

## Discussion

Chronic inflammation is a complex process that is involved in the development of many diseases: understanding the factors that can modulate it is of great importance in order to identify potential preventive or therapeutic strategies. In this study, we aimed to investigate the effects of BCG and influenza vaccination on the plasma inflammation proteome of healthcare workers who participated in the BCG‐CORONA trial. We also examined the interactions between these vaccinations and SARS‐CoV‐2 infection and sex.

We show that BCG vaccination is associated with a lower overall relative concentration of circulating inflammatory proteins in both men and women (compared to controls), albeit more strongly in men. This is in agreement with previous studies demonstrating reduced systemic inflammation after BCG vaccination.^14^, ^22^, ^23^ Furthermore, we show that quadrivalent inactivated influenza vaccination is also associated with lower overall relative concentrations of the assessed inflammation panel proteins in men, but not in women. This is also in agreement with a previous study.^24^ It is well established that immune regulation is influenced by sex‐specific pathways, as highlighted by ter Horst *et al*.^25^ We hypothesise that the more pronounced decrease in circulating inflammatory proteins observed in men after BCG or influenza vaccination may be attributed to the higher baseline systemic inflammation that is present in men compared to women. Conversely, women who naturally exhibit lower systemic inflammation are less likely to respond with further reduction in inflammatory markers.

This sex‐dependent difference in the response to vaccines highlights the influence of sex‐specific pathways in immune regulation and vaccine‐induced immune responses.

While both BCG and influenza vaccination were associated with lower relative concentrations of circulating inflammatory markers in men, it is important to recognise that the proteome profiles differ for the two vaccines, with some notable overlap (e.g. STAMBP and TNFSF14). We hypothesise that each vaccine has nuanced and specific effects on the inflammatory proteome. We speculate that the effects are mediated primarily through innate immune cells, such as monocytes, macrophages and NK cells, as these are the cells that are responsible for non‐specific effects of vaccines, such as BCG.^26^

The selective nature of the targeted proteomics approach (the selection of analytes in the Olink Inflammation panel is based on expert curation) prevented us from doing unbiased pathway enrichment analyses that may point towards the responsible cell types. However, the Human Protein Atlas (proteinatlas.org)^27^ reports that prominently affected proteins, such as MMP‐1 (Figure 1a) and CXCL11 (Figure 3a) are expressed more strongly in the innate immune system than the adaptive immune system, lending some credibility to this thought. Further investigations using more comprehensive proteome analyses would be able to unravel the exact immune pathways that are involved in the observed effects.

**Figure 5:**
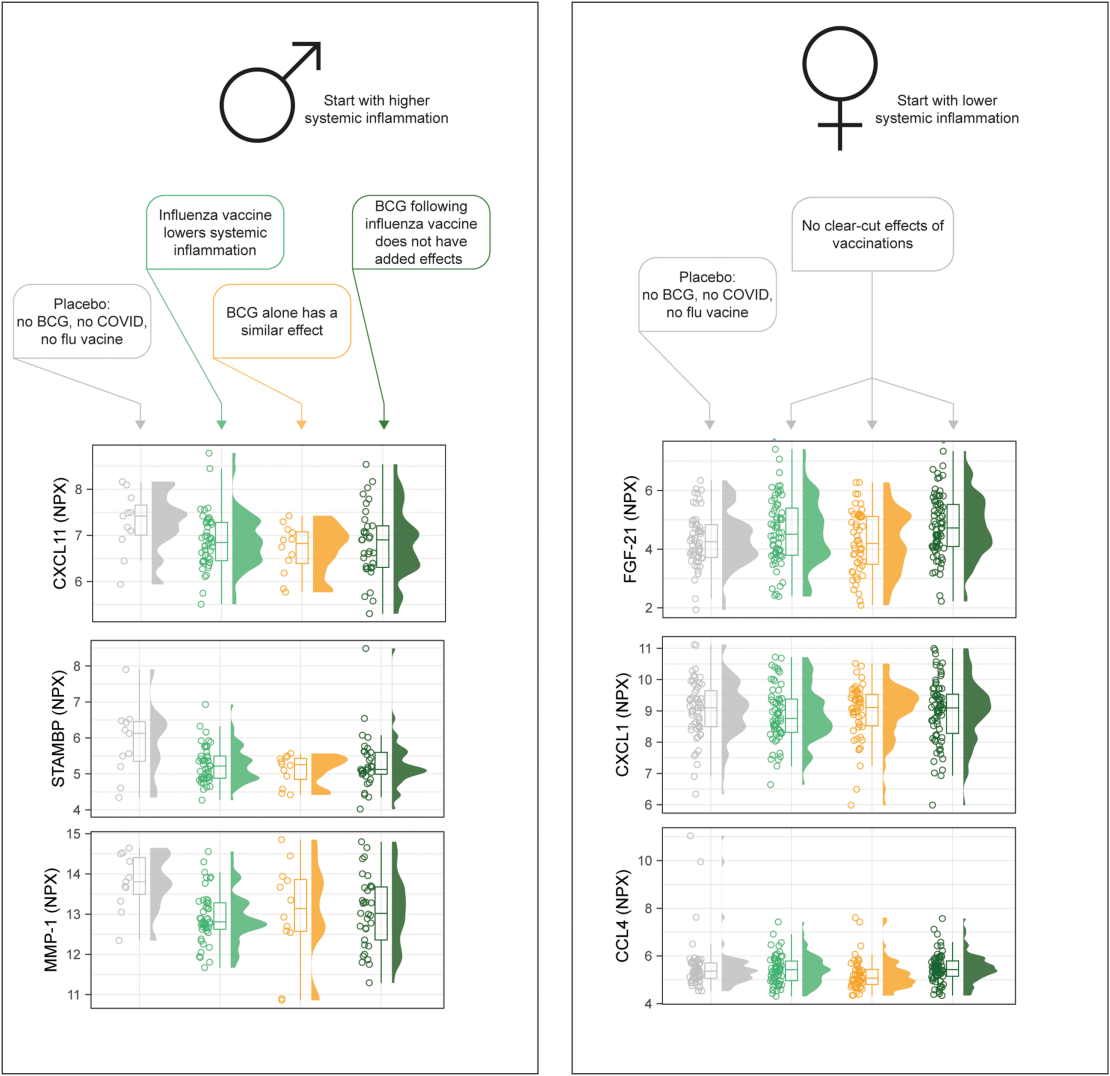
Summary of the interplay between influenza vaccine, Bacille Calmette–Guérin (BCG) vaccine and systemic inflammation in men (left) and women (right).

We describe for the first time the interaction between BCG and influenza vaccination at the level of the plasma inflammation proteome. Men—but not women—who received an influenza vaccination 6–8 months prior to randomisation had lower concentrations of the assessed inflammation panel proteins, but this overall relative concentration did not differ between the subgroups of men who had subsequently received BCG or placebo vaccination. We hypothesise that different vaccines may reduce inflammation, but that the individual proteins involved, and the extent of the reduction, depend on the specific vaccine, and that the effects plateau if multiple vaccinations are given within months of one another. We have illustrated this interaction in Figure 5.

Furthermore, previous studies on the effects of influenza vaccination on immune modulation have primarily focused on short‐term effects (until 6 weeks).^24^ Our data suggest that influenza vaccination leads to a sustained reduction in the relative concentrations of some inflammatory proteins for at least 6–8 months. To our knowledge, this is the first study that describes associations between influenza vaccination (or any vaccine) and decreased plasma inflammation proteins 6–8 months after vaccination. Our findings are in line with existing knowledge on the effects of trained immunity‐inducing vaccines on plasma inflammation proteins and could indicate that these effects last beyond the timeframe studied by the aforementioned longitudinal studies.

Having experienced a SARS‐CoV‐2 infection between randomisation and plasma sampling was associated with a trend towards lower systemic inflammation in the placebo group. However, this finding was not robust because the number of infections was low. In contrast, this trend was not seen in the BCG group. We hypothesise a potential interplay between a SARS‐CoV‐2‐induced dampening of inflammation and a BCG‐induced rescue effect, potentially promoting a more balanced immune response during viral infection. The majority of post‐COVID‐19 proteome studies show a similar trend of lower concentrations of CXCL10 and CXCL9 after infection,^28^ even in long‐COVID‐19 patients.^29^ However, in hospitalised patients—including those who are admitted to the Intensive Care Unit—these proteins are significantly upregulated.^30^, ^31^ This suggests that CXCL10 and CXCL9 are associated with disease severity and could potentially be used as biomarkers for disease severity or as targets for future therapies.

Individuals who reported respiratory symptoms during the 9‐month follow‐up after plasma sampling had slightly higher relative concentrations of CXCL11 and OPG at the time of plasma sampling. We hypothesise that individuals with low‐grade systemic inflammation may develop respiratory symptoms more easily than individuals without such inflammation. Purposefully designed longitudinal studies are required to identify individual inflammation proteins that are predictive for severe respiratory disease after exposure to a pathogen or allergen.

Our study had several limitations. One of the challenges that we encountered was the low number of SARS‐CoV‐2 infections in the first 12 weeks of the study, likely because of pandemic control measures, such as lockdowns and social distancing, during that period. Most SARS‐CoV‐2 infections in the trial took place during later epidemic peaks. This hindered our ability to perform a comprehensive analysis of the proteome signature induced by SARS‐CoV‐2 infection within 12 weeks of BCG/placebo vaccination. Additionally, certain subgroup analyses suffered from limited statistical power because of the relatively low number of male participants compared to female participants in the BCG‐CORONA trial. We therefore treated this study as exploratory and hypothesis‐generating and did not correct for multiple testing throughout the manuscript. This makes it likely that there are false‐positive cases of individual proteins that we label statistically significant, although it seems improbable that the observed overall shifts/patterns that are the focus of this study are solely a result of chance. Another limitation is that while the original trial was a randomised controlled trial for comparing the effects of BCG and placebo on SARS‐CoV‐2 incidence, these secondary analyses on a subpopulation of the overall trial population break the randomisation. We cannot therefore completely exclude some biases in case of influenza vaccination: for example, socioeconomic factors can influence both healthcare‐seeking behaviour (i.e. the likelihood of having received the influenza vaccine) and general health (which might be reflected in the levels of circulating inflammatory markers). The cohort size also does not allow for extended sub‐group analyses based on various host characteristics. It is also important to acknowledge that the current study was cross‐sectional in nature, making it difficult to establish causality. Our study focuses on plasma inflammatory proteins, which, while representative of systemic inflammation, may not precisely mirror the full complexity of the systemic inflammatory response. In view of these limitations, well‐controlled or even randomised, adequately powered longitudinal studies with repeated protein assessments are indispensable to confirm our findings.

In conclusion, our study suggests long‐lasting alterations in the plasma inflammation proteome associated with BCG vaccination, influenza vaccination and SARS‐CoV‐2 infection, and describes sex‐specific variations and interactions in these responses. Additionally, we provide potential leads for predictive biomarkers associated with respiratory symptoms in our study and with COVID‐19 severity in other studies.

After the necessary follow‐up studies to independently confirm our findings, we propose to investigate whether trained immunity‐inducing vaccines can be used to prophylactically reduce plasma inflammatory proteins. In addition to the known non‐specific protection against infections, these vaccines may thereby contribute to the prevention of diseases that are associated with chronic inflammation, such as cardiometabolic diseases (e.g. atherosclerosis, type 2 diabetes and metabolic syndrome) and neurodegenerative diseases (e.g. Alzheimer's disease). Considering recent developments across the fields of vaccinology and innate immunity, it is becoming increasingly clear that vaccine‐induced heterologous effects can have multifaceted effects on overall health and warrant further research.

## Methods

### Study design and subjects

This study was performed in a subgroup of the BCG‐CORONA trial participants (clinicaltrials.gov identifier: NCT04328441). This subgroup was composed of 419 adult healthcare workers who were included in the Radboud University Medical Center, Nijmegen, the Netherlands (one of the trial centres). They were randomly assigned to receive either BCG or placebo vaccination at the start of the COVID‐19 epidemic in March/April 2020 (for further details on study methods, refer to 10 Doesschate *et al*.^20^). Twelve weeks after randomisation, blood samples were collected from 363 participants (86.6% of randomised population from this site attending the sampling visit). These samples were used to measure SARS‐CoV‐2 serology and inflammatory proteins in plasma using the Olink proximity extension assay technology inflammation panel.

Following quality control of the data, the plasma inflammation proteome of 357 subjects (98.3% of the sampled population) was analysed, of whom 176 had received a placebo vaccination and 181 a BCG vaccination. All participants (see also Table 1) completed questionnaires regarding their health via a mobile phone diary application during follow‐up, as described previously.^20^ Leveraging this information, we also determined which participants had received the influenza vaccine (quadrivalent inactivated) in the winter immediately preceding the COVID‐19 epidemic and who developed symptoms of respiratory infection in the 9 months following plasma sampling. Of note, in our hospital, the influenza vaccination campaign ran from mid‐November 2019 until mid‐December 2019, and the plasma collection for this study was performed in July 2020. Respiratory symptoms were defined as the presence of cough or dyspnoea with a severity score of ≥ 2 (mild symptoms), or the presence of nasal congestion or sore throat with a severity score of ≥ 3 (moderate symptoms). A SARS‐CoV‐2 episode was defined as having positive SARS‐CoV‐2 serology at the time of sampling; it was assumed that all participants had their episode after BCG/placebo vaccination. Combining these data allowed the formation of four main comparison groups: individuals who received BCG only, those who received the influenza vaccine only, as well as those who received both BCG and influenza vaccines, and a control group (BCG and influenza vaccine naïve); comparisons between these groups were done in those seronegative for SARS‐CoV‐2 at the time of sampling. Two additional comparisons were made based on SARS‐CoV‐2 positivity and respiratory symptoms, irrespective of influenza vaccination status in BCG and placebo recipients. Supplementary figure 2 shows a visual representation of the study's structure and timeline.

### Targeted proteomics analyses

All samples were sent to Olink (Sweden) for targeted proteomics analysis using their proximity extension assay technology. For more information on this technology, including assay validation and precision, please refer to the company website (https://olink.com/our‐platform/our‐pea‐technology/). The relative concentrations of an expert‐curated panel of 92 proteins related to the immune system (both pro‐ and anti‐inflammatory proteins are included that are related to different kinds of immune processes) were measured (Olink inflammation panel), of which 69 were retained for analysis after filtering out proteins that were detected in < 33% of the study samples (this cut‐off was arbitrarily chosen; Supplementary figure 3). After quality control, 357 samples (98.3%) were included in the final analyses. We compared different subgroups (Supplementary figure 2) of our study population using the Wilcoxon rank sum test. Volcano plots were created using the Enhanced Volcano R package.

### Statistical analysis and software

The Olink Normalized Protein eXpression (NPX) value is an arbitrary unit on a log_2_‐scale, where a high NPX value corresponds to a high protein concentration. The difference in average NPX values between two groups corresponds to the log_2_ fold difference between comparison groups (log_2_FD): log_2_(*x/y*) = log_2_(*x*)−log_2_(*y*). For this study, the difference in median NPX was chosen to calculate log_2_FD. The relative concentration of proteins was considered higher or upregulated if the log_2_FD between comparison groups was above 0. Proteins were considered to be lower or downregulated if the log_2_FD was below 0. Note that all comparisons were cross‐sectional and log_2_FDs therefore do not refer to temporal cause‐effect relationships. Unadjusted *P*‐values for each protein were calculated using the Wilcoxon rank sum test. To determine whether the median log_2_FD of all 69 proteins combined was significantly different from 0 (indicating a major shift in overall levels of inflammatory proteins in the circulation), the one‐sample Wilcoxon rank sum test was used.

Linear regression analysis (using the base R stats package) was performed for the selected proteins during our evaluation of the effects of BCG and influenza, using the NPX value as a continuous outcome variable and age, sex and vaccine exposures as independent predictor variables. Exact model formulas are indicated together with the model results.

Throughout this manuscript, unadjusted two‐tailed *P*‐values below 0.05 were considered statistically significant. Since this is an explorative study, we report crude *P*‐values without adjustment for multiple testing.

All analyses were performed in R (4.2.1) using the following packages: Tidyverse^32^, ^33^ core packages (v2.0.0), janitor^34^ (v2.2.0), OlinkAnalyze^35^ (v3.3.1), broom^36^ (v1.0.4), EnhancedVolcano^37^ (v1.14.0), ggrepel^38^ (v0.9.3), ggdist^39^ (v3.2.1), gghalves^40^ (v0.1.4), patchwork^41^ (v1.1.2), readxl^42^ (v1.4.2), gridExtra^43^ (v2.3) and ggtext^44^ (v0.1.2). Figures were exported from R using the patchwork package and finalised in Adobe Illustrator.

## Author contributions

**Priya A Debisarun:** Conceptualization; data curation; formal analysis; investigation; methodology; project administration; resources; software; visualization; writing – original draft; writing – review and editing. **Rutger J Röring:** Data curation; formal analysis; funding acquisition; investigation; methodology; resources; software; validation; visualization; writing – original draft; writing – review and editing. **Özlem Bulut:** Investigation; validation. **Thijs ten Doesschate:** Data curation; software. **Thomas W van der Vaart:** Data curation; software. **Vinod Kumar:** Methodology; writing – review and editing. **Heidi Dijkstra:** Investigation. **Helga Lemmers:** Investigation. **Axel B Janssen:** Data curation. **Karin Veerman:** Conceptualization. **Rob ter Heine:** Investigation; resources; writing – review and editing. **Reinout van Crevel:** Conceptualization; writing – review and editing. **Jaap ten Oever:** Conceptualization; project administration; writing – review and editing. **Leo AB Joosten:** Conceptualization; methodology. **Marc J Bonten:** Conceptualization; writing – review and editing. **Cornelis H van Werkhoven:** Conceptualization; writing – review and editing. **Janneke HHM van de Wijgert:** Conceptualization; data curation; funding acquisition; methodology; resources; writing – review and editing. **Mihai G Netea:** Conceptualization; funding acquisition; methodology; project administration; resources; supervision; writing – original draft; writing – review and editing.

## Conflict of interest

MGN is the scientific founder of TTxD, Biotrip and Lemba Therapeutics and has obtained research grants from GSK Biologicals, TTxD and Ono Pharma, as well as consultancy fees from TTxD. LABJ is the scientific founder of TTxD and Lemba Therapeutics. CHvW reports grants from DaVolterra, bioMérieux and LimmaTech, as well as consultancy fees from Merck/MSD and Sanofi‐Pasteur.

## Supporting information


Supplementary figures 1‐3

## Data Availability

Data are available from the corresponding author upon reasonable request.

## References

[1] Sharif S , Van der Graaf Y , Cramer MJ *et al*. Low‐grade inflammation as a risk factor for cardiovascular events and all‐cause mortality in patients with type 2 diabetes. Cardiovasc Diabetol 2021; 20: 220. 34753497 10.1186/s12933-021-01409-0 PMC8579639

[2] Esser N , Legrand‐Poels S , Piette J , Scheen AJ , Paquot N . Inflammation as a link between obesity, metabolic syndrome and type 2 diabetes. Diabetes Res Clin Pract 2014; 105: 141–150. 24798950 10.1016/j.diabres.2014.04.006

[3] Hajer GR , van Haeften TW , Visseren FL . Adipose tissue dysfunction in obesity, diabetes, and vascular diseases. Eur Heart J 2008; 29: 2959–2971. 18775919 10.1093/eurheartj/ehn387

[4] Michaud M , Balardy L , Moulis G *et al*. Proinflammatory cytokines, aging, and age‐related diseases. J Am Med Dir Assoc 2013; 14: 877–882. 23792036 10.1016/j.jamda.2013.05.009

[5] Mou Y , Du Y , Zhou L *et al*. Gut microbiota interact with the brain through systemic chronic inflammation: implications on neuroinflammation, neurodegeneration, and aging. Front Immunol 2022; 13: 796288. 35464431 10.3389/fimmu.2022.796288 PMC9021448

[6] World Health Organization . How do vaccines work. WHO 2020.

[7] Tanner AR , Dorey RB , Brendish NJ , Clark TW . Influenza vaccination: protecting the most vulnerable. Eur Respir Rev 2021; 30: 200258. 33650528 10.1183/16000617.0258-2020 PMC9488965

[8] Aaby P , Ravn H , Fisker AB , Rodrigues A , Benn CS . Is diphtheria‐tetanus‐pertussis (DTP) associated with increased female mortality? A meta‐analysis testing the hypotheses of sex‐differential non‐specific effects of DTP vaccine. Trans R Soc Trop Med Hyg 2016; 110: 570–581. 27856947 10.1093/trstmh/trw073 PMC5155548

[9] Blok BA , Arts RJ , van Crevel R , Benn CS , Netea MG . Trained innate immunity as underlying mechanism for the long‐term, nonspecific effects of vaccines. J Leukoc Biol 2015; 98: 347–356. 26150551 10.1189/jlb.5RI0315-096R

[10] Arts RJW , Carvalho A , La Rocca C *et al*. Immunometabolic pathways in BCG‐induced trained immunity. Cell Rep 2016; 17: 2562–2571. 27926861 10.1016/j.celrep.2016.11.011 PMC5177620

[11] Arts RJW , Moorlag S , Novakovic B *et al*. BCG vaccination protects against experimental viral infection in humans through the induction of cytokines associated with trained immunity. Cell Host Microbe 2018; 23: 89–100 e105. 29324233 10.1016/j.chom.2017.12.010

[12] Kleinnijenhuis J , Quintin J , Preijers F *et al*. Bacille Calmette‐Guerin induces NOD2‐dependent nonspecific protection from reinfection via epigenetic reprogramming of monocytes. Proc Natl Acad Sci USA 2012; 109: 17537–17542. 22988082 10.1073/pnas.1202870109 PMC3491454

[13] Kleinnijenhuis J , Quintin J , Preijers F *et al*. Long‐lasting effects of BCG vaccination on both heterologous Th1/Th17 responses and innate trained immunity. J Innate Immun 2014; 6: 152–158. 24192057 10.1159/000355628 PMC3944069

[14] Koeken VA , de Bree LCJ , Mourits VP *et al*. BCG vaccination in humans inhibits systemic inflammation in a sex‐dependent manner. J Clin Invest 2020; 130: 5591–5602. 32692728 10.1172/JCI133935 PMC7524503

[15] World Health Organization . History of the influenza vaccine: a year‐round disease affecting everyone. WHO 2023. https://www.who.int/news-room/spotlight/history-of-vaccination/history-of-influenza-vaccination

[16] Beigel JH , Tomashek KM , Dodd LE *et al*. Remdesivir for the treatment of Covid‐19 ‐ final report. N Engl J Med 2020; 383: 1813–1826. 32445440 10.1056/NEJMoa2007764 PMC7262788

[17] Group RC . Tocilizumab in patients admitted to hospital with COVID‐19 (RECOVERY): a randomised, controlled, open‐label, platform trial. Lancet 2021; 397: 1637–1645. 33933206 10.1016/S0140-6736(21)00676-0 PMC8084355

[18] Pittet LF , Messina NL , Orsini F *et al*. Randomized trial of BCG vaccine to protect against Covid‐19 in health care workers. N Engl J Med 2023; 388: 1582–1596. 37099341 10.1056/NEJMoa2212616 PMC10497190

[19] Polack FP , Thomas SJ , Kitchin N *et al*. Safety and efficacy of the BNT162b2 mRNA Covid‐19 vaccine. N Engl J Med 2020; 383: 2603–2615. 33301246 10.1056/NEJMoa2034577 PMC7745181

[20] Ten Doesschate T , van der Vaart TW , Debisarun PA *et al*. Bacillus Calmette‐Guerin vaccine to reduce healthcare worker absenteeism in COVID‐19 pandemic, a randomized controlled trial. Clin Microbiol Infect 2022; 28: 1278–1285. 35489606 10.1016/j.cmi.2022.04.009 PMC9046133

[21] Claus J , Ten Doesschate T , Gumbs C *et al*. BCG vaccination of health care workers does not reduce SARS‐CoV‐2 infections nor infection severity or duration: a randomized placebo‐controlled trial. MBio 2023; 14: e0035623. 36976004 10.1128/mbio.00356-23 PMC10128007

[22] Koeken V . Controlling inflammation in the elderly with BCG vaccination. Sci Adv 2021; 7: abk1668. 10.1126/sciadv.abk1668 PMC8336952 34348906

[23] Pavan Kumar N , Padmapriyadarsini C , Rajamanickam A *et al*. Effect of BCG vaccination on proinflammatory responses in elderly individuals. Sci Adv 2021; 7: eabg7181. 34348897 10.1126/sciadv.abg7181 PMC8336950

[24] Debisarun PA , Gossling KL , Bulut O *et al*. Induction of trained immunity by influenza vaccination ‐ impact on COVID‐19. PLoS Pathog 2021; 17: e1009928. 34695164 10.1371/journal.ppat.1009928 PMC8568262

[25] Ter Horst R , van den Munckhof ICL , Schraa K *et al*. Sex‐specific regulation of inflammation and metabolic syndrome in obesity. Arterioscler Thromb Vasc Biol 2020; 40: 1787–1800. 32460579 10.1161/ATVBAHA.120.314508 PMC7310302

[26] Netea MG , Domínguez‐Andrés J , Barreiro LB *et al*. Defining trained immunity and its role in health and disease. Nat Rev Immunol 2020; 20: 375–388. 32132681 10.1038/s41577-020-0285-6 PMC7186935

[27] Karlsson M , Zhang C , Méar L *et al*. A single–cell type transcriptomics map of human tissues. Sci Adv 2021; 7: eabh2169. 34321199 10.1126/sciadv.abh2169 PMC8318366

[28] Zoodsma M , de Nooijer AH , Grondman I *et al*. Targeted proteomics identifies circulating biomarkers associated with active COVID‐19 and post‐COVID‐19. Front Immunol 2022; 13: 1027122. 36405747 10.3389/fimmu.2022.1027122 PMC9670186

[29] Zhao J , Schank M , Wang L *et al*. Plasma biomarkers for systemic inflammation in COVID‐19 survivors. Proteomics Clin Appl 2022; 16: e2200031. 35929818 10.1002/prca.202200031 PMC9539278

[30] Filbin MR , Mehta A , Schneider AM *et al*. Longitudinal proteomic analysis of severe COVID‐19 reveals survival‐associated signatures, tissue‐specific cell death, and cell–cell interactions. Cell Rep Med 2021; 2: 100287. 33969320 10.1016/j.xcrm.2021.100287 PMC8091031

[31] Arunachalam PS , Wimmers F , Mok CKP *et al*. Systems biological assessment of immunity to mild versus severe COVID‐19 infection in humans. Science 2020; 369: 1210–1220. 32788292 10.1126/science.abc6261 PMC7665312

[32] Wickham H . tidyverse: easily install and load the tidyverse. 2023. https://tidyverse.tidyverse.org/

[33] Wickham H , Averick M , Bryan J *et al*. Welcome to the tidyverse. J Open Source Softw 2019; 4(43): 1686.

[34] Firke S . janitor: simple tools for examining and cleaning dirty data. 2023. doi: 10.32614/CRAN.package.janitor

[35] Nevola K , Sandin M , Guess J . OlinkAnalyze: facilitate analysis of proteomic data from olink. 2023. doi: 10.32614/CRAN.package.OlinkAnalyze

[36] Robinson D , Hayes A , Couch S . broom: convert statistical objects into tidy tibbles. 2023. doi: 10.32614/CRAN.package.broom

[37] Blighe K , Rana S , Lewis M . EnhancedVolcano: publication‐ready volcano plots with enhanced colouring and labeling. 2022. doi:10.18129/B9.bioc.EnhancedVolcano

[38] Slowikowski K . ggrepel: automatically position non‐overlapping text labels with ggplot2. 2023. doi: 10.32614/CRAN.package.ggrepel

[39] Kay M . ggdist: visualizations of distributions and uncertainty. 2023. doi: 10.32614/CRAN.package.ggdist 37883271

[40] Tiedemann F . gghalves: Compose Half‐Half Plots Using Your Favourite Geoms. 2022. doi: 10.32614/CRAN.package.gghalves

[41] Pedersen TL . patchwork: the composer of plots. 2022. doi: 10.32614/CRAN.package.patchwork

[42] Wickham H , Bryan J . readxl: read excel files. 2023. doi: 10.32614/CRAN.package.readxl

[43] Auguie B . gridExtra: miscellaneous functions for “Grid” graphics. 2017. doi: 10.32614/CRAN.package.gridExtra

[44] Wilke CO , Wiernik BM . ggtext: improved text rendering support for ggplot2. 2022. doi: 10.32614/CRAN.package.ggtext

