# Supplementary material for: Long‐term effects of influenza and Bacille Calmette–Guérin vaccination on systemic inflammation
Source: Clin Transl Immunology. 2025 Sep 11;14(9):e70047. doi: 10.1002/cti2.70047 (PMC12423592; doi:10.1002/cti2.70047)
Supplement: Supplementary file 1 — Supplementary figures 1‐3 [file CTI2-14-e70047-s001.pdf]

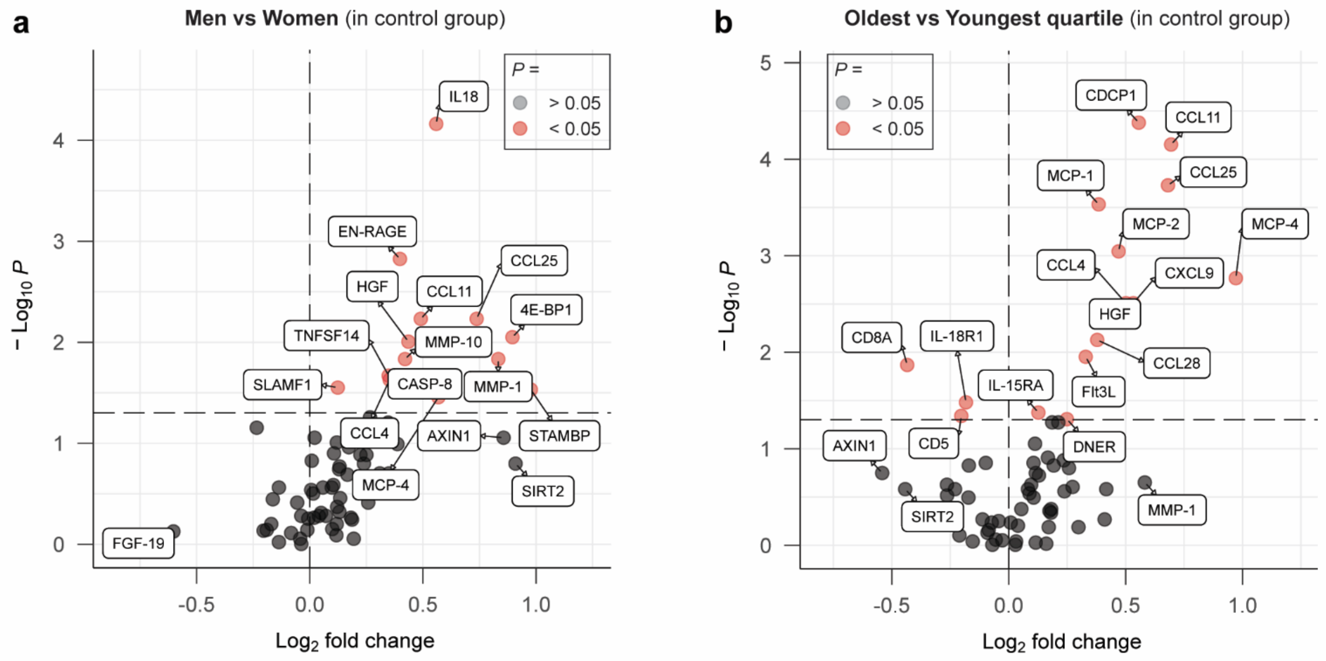

Supplementary figure 1: Differences in circulating inflammatory proteins based on sex and age, in those who had not received influenza vaccins or BCG. (**a**) Differences between men and women. (**b**) Differences between the oldest and youngest quartile.

a

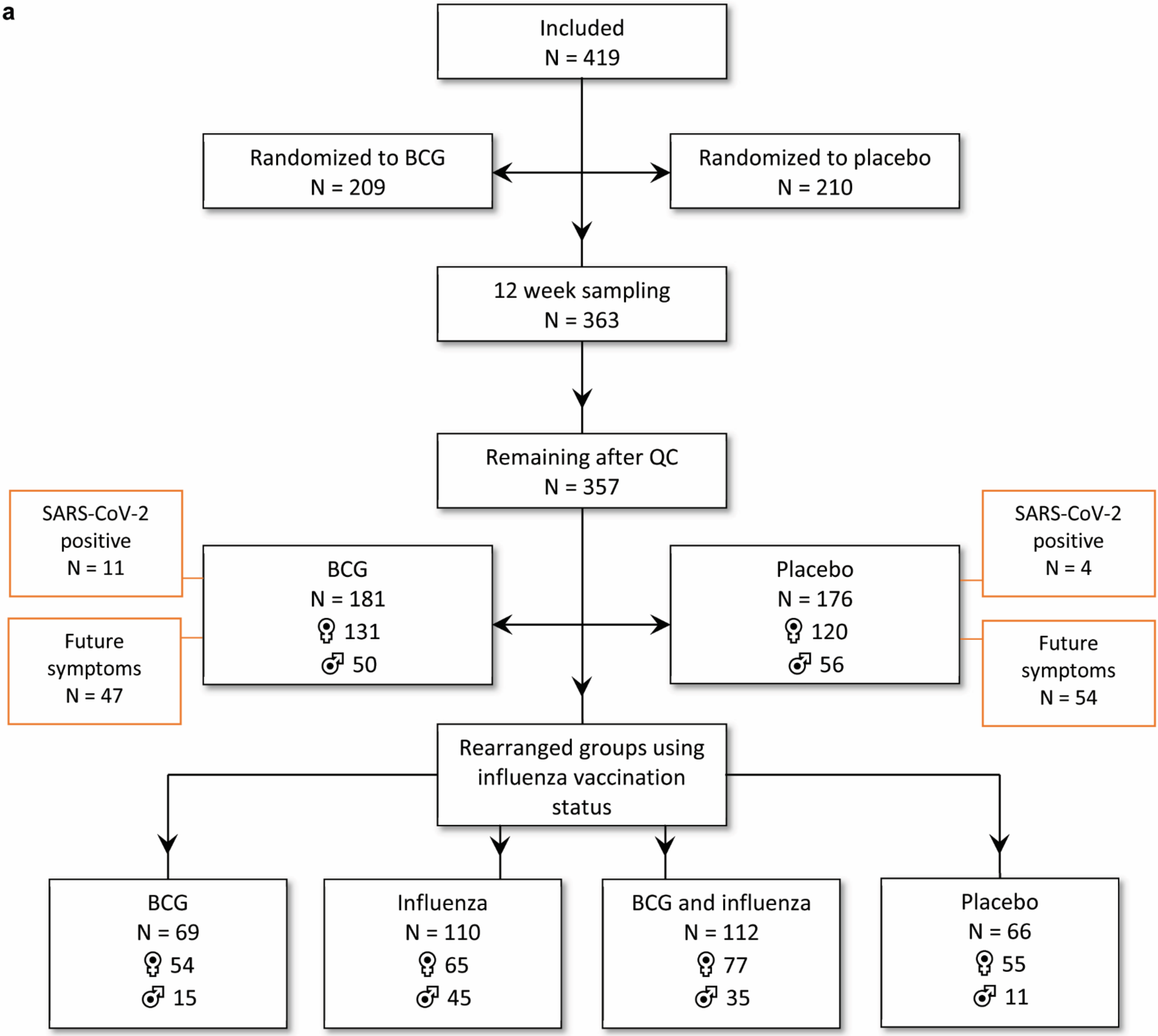

Supplementary figure 2: Flow chart showing the original study design and the subgroups used for this study.

a

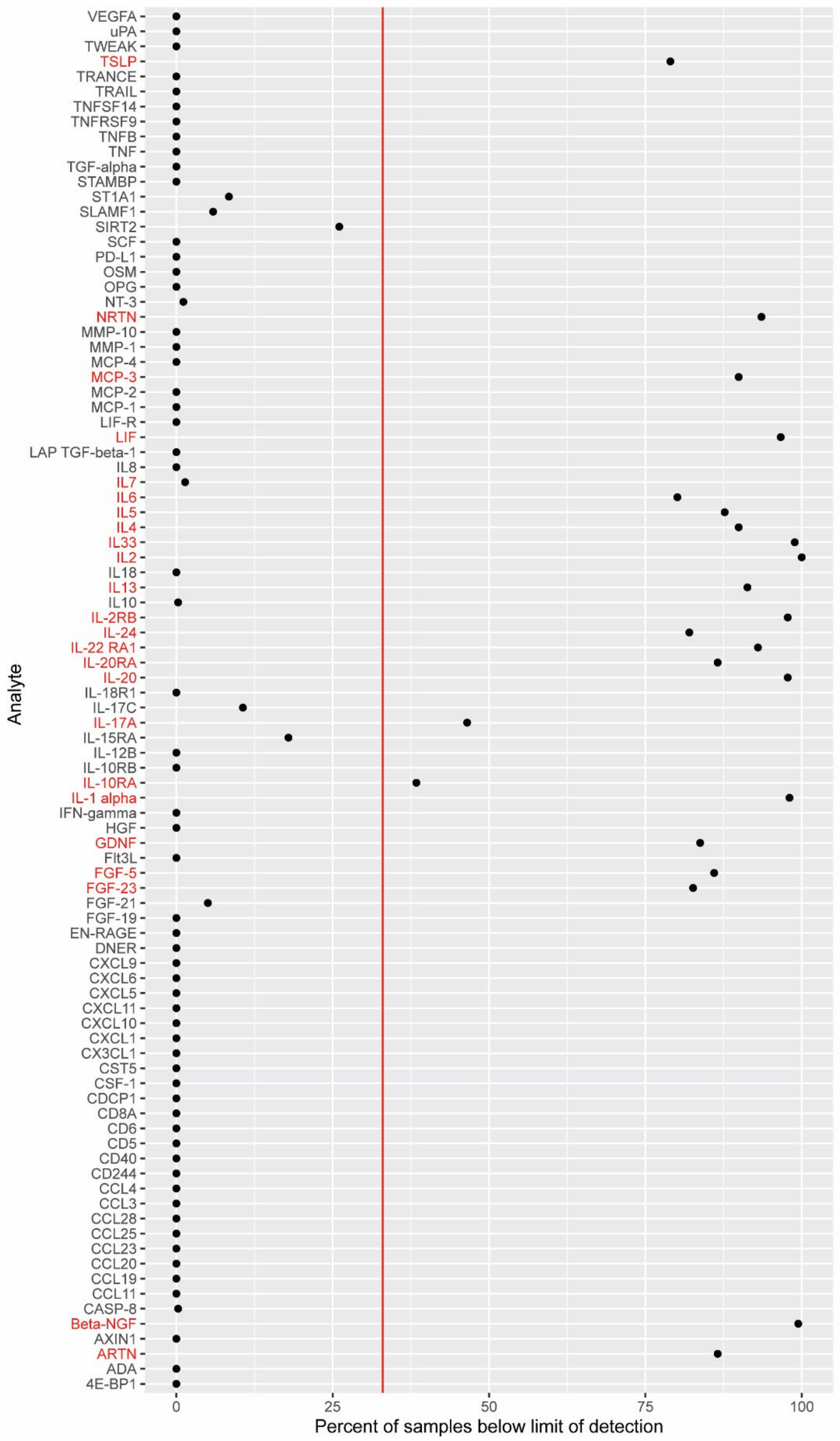

Supplementary Figure 3: Selection of proteins for analysis based on percentage of samples under the limit of detection. Samples in red were excluded from the analyses.
